# Silica-Supported Titania–Zirconia Nanocomposites: Structural and Morphological Characteristics in Different Media

**DOI:** 10.1186/s11671-016-1304-1

**Published:** 2016-02-29

**Authors:** Iryna Sulym, Olena Goncharuk, Dariusz Sternik, Ewa Skwarek, Anna Derylo-Marczewska, Wladyslaw Janusz, Vladimir M. Gun’ko

**Affiliations:** Chuiko Institute of Surface Chemistry, NASU, 17 General Naumov Str., 03164 Kyiv, Ukraine; Faculty of Chemistry, Maria Curie-Sklodowska University, 20-031 Lublin, Poland

**Keywords:** Nanocomposites, TiO_2_–ZrO_2_/SiO_2_, Phase composition, Nanocrystallinity, Particle size distribution

## Abstract

A series of TiO_2_–ZrO_2_/SiO_2_ nanocomposites were synthesized using a liquid-phase method and characterized by various techniques, namely, nitrogen adsorption–desorption, X-ray diffraction (XRD), X-ray photoelectron spectroscopy (XPS), Raman spectroscopy, high-resolution transmission electron microscopy, and photon correlation spectroscopy (PCS). It was revealed that the component ratio and calcination temperature affect the phase composition of nanocomposites. Composites TiZrSi1 (TiO_2_:ZrO_2_:SiO_2_ = 3:10:87) and TiZrSi2 (10:10:80) calcined at 1100 °С demonstrate the presence of *t*-ZrO_2_ crystallites in TiZrSi1 and ZrTiO_4_ phase in TiZrSi2. The samples calcined at 550 °С were amorphous as it was found from XRD data. According to the Raman spectra, the bands specific for anatase are observed in TiZrSi2. According to XPS data, Zr and Ti are in the highest oxidation state (+4). Textural analysis shows that initial silica is mainly meso/macroporous, but composites are mainly macroporous. The particle size distributions in aqueous media showed a tendency of increasing particle size with increasing TiO_2_ content in the composites.

## Background

Highly disperse (nanoparticulate) oxide composites are of great interest for individual applications not only as heterogeneous catalysts with an adjustable set and strength of surface active sites [1–4] but also as a part of organic–inorganic composites and polymer fillers [5, 6]. Combination of dissimilar oxides allows to create surface active sites, which are absent in individual components [7]. The nature of active sites of solid acid catalysts is defined by mobile surface protons generating Brønsted acid sites and coordinately unsaturated cationic centers as Lewis acid sites [8]. Therefore, much attention has been focused on development of binary or ternary metal oxides as heterogeneous catalysts [1]. Thus, the main objective to prepare such nanoscale systems is aimed at controlling their surface composition and particle morphology. One of the common methods of the synthesis of nanoparticulate oxides is based on the use of a substrate of a high specific surface area. The fumed silica properties are a convenient vehicle for the synthesis of the mentioned composites due to silica inertness in catalytic processes, developed surface area, and homogeneity of active sites on a surface [9]. Among various metal oxide catalysts, the combination of titania and zirconia has attracted attention in recent years. These mixed oxides have been extensively used as catalysts and catalyst supports for a wide variety of reactions [2]. TiO_2_–ZrO_2_ mixed oxide composites are used as photocatalysts due to a reduced bandgap in comparison to individual components [3, 10–15]. They have been reported to exhibit a high surface acidity due to an imbalance of charges resulting from the formation of the Ti–O–Zr bridges [14, 16]. According to [11], TiO_2_/SiO_2_ and TiO_2_/ZrO_2_ are characterized by more acidic properties than single/pure components. TiO_2_–ZrO_2_ system is a strong solid acid showing catalytic activity in such reactions as isomerization and cracking of alkanes, hydration and polymerization of alkenes, etc. [7, 17]. The most widely employed methods to prepare TiO_2_–ZrO_2_ composites are co-precipitation [18, 19] and sol–gel synthesis [2, 10, 20, 21]. A method of grafting of mixed oxides onto a surface of highly disperse matrices with nonporous nanoparticles can be a good alternative to the mentioned methods. Therefore, the objective of this study was the synthesis of silica-supported titania–zirconia nanocomposites (TiO_2_–ZrO_2_/SiO_2_) and investigation of their morphological and structural properties.

## Methods

### Materials

Fumed silica (pilot plant of the Chuiko Institute of Surface Chemistry, Kalush, Ukraine), zirconium (Aldrich, > 98 % Zr(acac)_4_), and titanyl (C_10_H_11_O_5_Ti) acetylacetonates (Merck) were used as precursors to prepare oxide composites.

### Synthesis of Silica-Supported Titania–Zirconia Nanocomposites

Silica-supported titania–zirconia nanocomposites (TiO_2_–ZrO_2_/SiO_2_) were prepared using a liquid-phase method. The synthesis was performed in a glass double-neck reactor equipped with a propeller agitator and a reflux condenser. Zr(acac)_4_ and C_10_H_11_O_5_Ti solutions in isopropyl alcohol (IPA) were added to fumed silica (5 g; previously calcined at 500 °C; specific surface area *S* = 283 m^2^/g) at 82.5 °С. The reaction mixture was stirred in the refluxing tube for 1 h. Then, IPA and acetylacetone were removed from the mixture by evacuation. The solid products were dried and calcined at 550 °С and 1100 °С for 1 h. According to [22], the temperature range 500–550 °C corresponds to the destruction of acetylacetonate ligands and complete removal of the volatile carbon components upon oxide formation. But at 550 °C, a high probability of the formation of the amorphous structure takes place, while the temperature of 1100 °C was chosen as sufficient for crystalline structure formation. The content of grafted TiO_2_ was varied from 3 to 10 wt.% while ZrO_2_ content was held constant at 10 wt.% (samples TiZrSi1 and TiZrSi2, respectively).

### X-Ray Powder Diffraction Analysis (XRD)

X-ray diffraction patterns were recorded at room temperature using a DRON-3M diffractometer (Burevestnik, St. Petersburg, Russia) with Cu *K*_*α*_ (*λ* = 0.15418 nm) radiation and a Ni filter in the 2*θ* range from 10° to 70°. The average size of nanocrystallites (*D*_cr_) was estimated according to the Scherrer equation [23]. Crystalline structure of samples was analyzed using the JCPDS Database (International Center for Diffraction Data, PA, 2001) [24]. Silica was totally amorphous in all samples.

### Raman Spectroscopy (RS)

The Raman spectra were recorded over the 150–3200-cm^−1^ range using an inVia Reflex Microscope DMLM Leica Research Grade, Reflex (Renishaw, UK), with Ar^+^ ion laser excitation at *λ*_0_ = 514.5 nm. For each sample, the spectra were recorded at several points in order to ascertain the homogeneity of the sample, and the averages of all these spectra were plotted.

### X-Ray Photoelectron Spectroscopy (XPS)

The XPS measurements were performed using a VG Scienta R4000 electron analyzer with an MX650 monochromatized Al *K*_*α*_ (1486.6 eV) radiation source. The binding energy (BE) was referenced to Si 2p (BE = 103.5 eV) with an accuracy of ±0.1 eV. Peak fitting was done using Casa XP5 with Shirley background and 10:90 Lorentzian/Gaussian convolution product shapes. The atomic concentration ratios were achieved by determining the elemental peak areas, following a Shirley background subtraction by the usual procedures documented in the literature [25].

### High-Resolution Transmission Electron Microscopy (HRTEM)

The particulate morphology was analyzed using high-resolution transmission electron microscope (HRTEM) employing a Tecna™ G2 T20 X-TWIN (FEI Company, USA) apparatus operating at a voltage of 200 kV with LaB6 electron source. The samples were supported on holey carbon copper grids by dropping ethanol suspensions containing uniformly dispersed oxide powders.

### Textural Characterization

To analyze the textural characteristics of TiO_2_–ZrO_2_/SiO_2_ nanocomposites, low-temperature (77.4 K) nitrogen adsorption–desorption isotherms were recorded using an automatic gas adsorption analyzer ASAP 2405N (Micromeritics Instrument Corp., USA) after outgassing the samples at 110 °C for 2 h in a vacuum chamber. The values of the specific surface area (*S*_BET_) were calculated according to the standard BET method [26]. The total pore volume *V*_p_ was evaluated by converting the volume of adsorbed nitrogen at *p/p*_0_ = 0.98–0.99 (*p* and *p*_0_ denote the equilibrium pressure and saturation pressures of nitrogen at 77.4 K, respectively) to the volume of liquid nitrogen per gram of adsorbent. The nitrogen desorption data were used to compute the pore size distributions (differential *f*_V_ ~ d*V*_p_/d*R* and *f*_S_ ~ d*S*/d*R*) using a self-consistent regularization (SCR) procedure under non-negativity condition (*f*_V_ ≥ 0 at any pore radius *R*) at a fixed regularization parameter *α* = 0.01 with voids (V) between spherical nonporous nanoparticles packed in random aggregates (V/SCR model) [27]. The differential pore size distributions with respect to pore volume *f*_V_ ~ d*V*/d*R*, *∫f*_V_d*R* ~ *V*_p_ were re-calculated to incremental pore size distributions (IPSD) at Φ_V_(*R*_*i*_) = (*f*_V_(*R*_*i*+1_) + *f*_V_(*R*_*i*_))(*R*_*i*+1_ − *R*_*i*_)/2 at ∑Φ_V_(*R*_*i*_) = *V*_p_). The *f*_V_ and *f*_S_ functions were also used to calculate contributions of micropores (*V*_micro_ and *S*_micro_ at 0.35 nm < *R* < 1 nm), mesopores (*V*_meso_ and *S*_meso_ at 1 nm < *R* < 25 nm), and macropores (*V*_macro_ and *S*_macro_ at 25 nm < *R* < 100 nm).

### Particle Size Distribution in Aqueous Media

Particle sizing for the aqueous suspensions of different fine oxides were carried out using a Zetasizer 3000 (Malvern Instruments) apparatus based on photon correlation spectroscopy (PCS, *λ* = 633 nm, *Θ* = 90°, software version 1.3).

The aqueous suspensions of oxides 0.1 wt.% were prepared using an ultrasonic disperser for 5 min (Sonicator Misonix Inc., power 500 W and frequency 22 kHz) prior to measuring particle size distribution.

## Discussion

### Textural Characterization

**Fig. 1:**
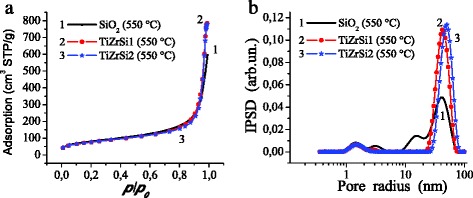
Nitrogen adsorption–desorption isotherms (**a**) and incremental pore size distributions (**b**) for initial silica (*curve 1*), TiZrSi1 (*2*), and TiZrSi2 (*3*) calcined at 550 °C

The nitrogen adsorption–desorption isotherms obtained for initial silica and composites (Fig. 1a) demonstrate sigmoidal-shaped behavior with a narrow hysteresis loop. The incremental pore (voids between particles in aggregates) size distribution functions (Fig. 1b) show that the textural characteristics change after the modification.

The specific surface area (Table 1, *S*_BET_) does not demonstrate a significant reduction after grafting of titania/zirconia. However, the total pore volume increases for TiO_2_–ZrO_2_/SiO_2_ compared to the initial silica. Furthermore, there is a significant decrease in mesopore contribution to the total porosity with a simultaneous increase in contribution of macropores. Moreover, the microporosity is slightly reduced for composites compared to the initial silica. Thus, the analysis of the results suggests the existence of mainly meso/macroporosity of aggregates of the initial silica and mainly macroporosity of the composites (Fig. 1b).

**Table 1:** Textural characteristics of initial and titania–zirconia-coated silica

Sample	*S*_BET_ (m^2^/g)	*S*_micro_ (m^2^/g)	*S*_meso_ (m^2^/g)	*S*_macro_ (m^2^/g)	*V*_micro_ (cm^3^/g)	*V*_meso_ (cm^3^/g)	*V*_macro_ (cm^3^/g)	*V*_p_ (cm^3^/g)	*R*_p,*V*_ (nm)
SiO_2_	283	21	225	38	0.008	0.35	0.57	0.93	29
TiZrSi1	276	17	163	97	0.005	0.08	1.12	1.21	39
TiZrSi2	280	18	169	92	0.005	0.07	1.13	1.20	45

Specific surface area in total (*S*_BET_), of nanopores (*S*_micro_), mesopores (*S*_meso_), macropores (*S*_macro_), and respective specific pore volumes (*V*_p_, *V*_micro_, *V*_meso_, *V*_macro_). *R*_p,*V*_ represents the average pore radius determined from the differential pore size distributions with respect to the pore volume

### High-Resolution Transmission Electron Microscopy

**Fig. 2:**
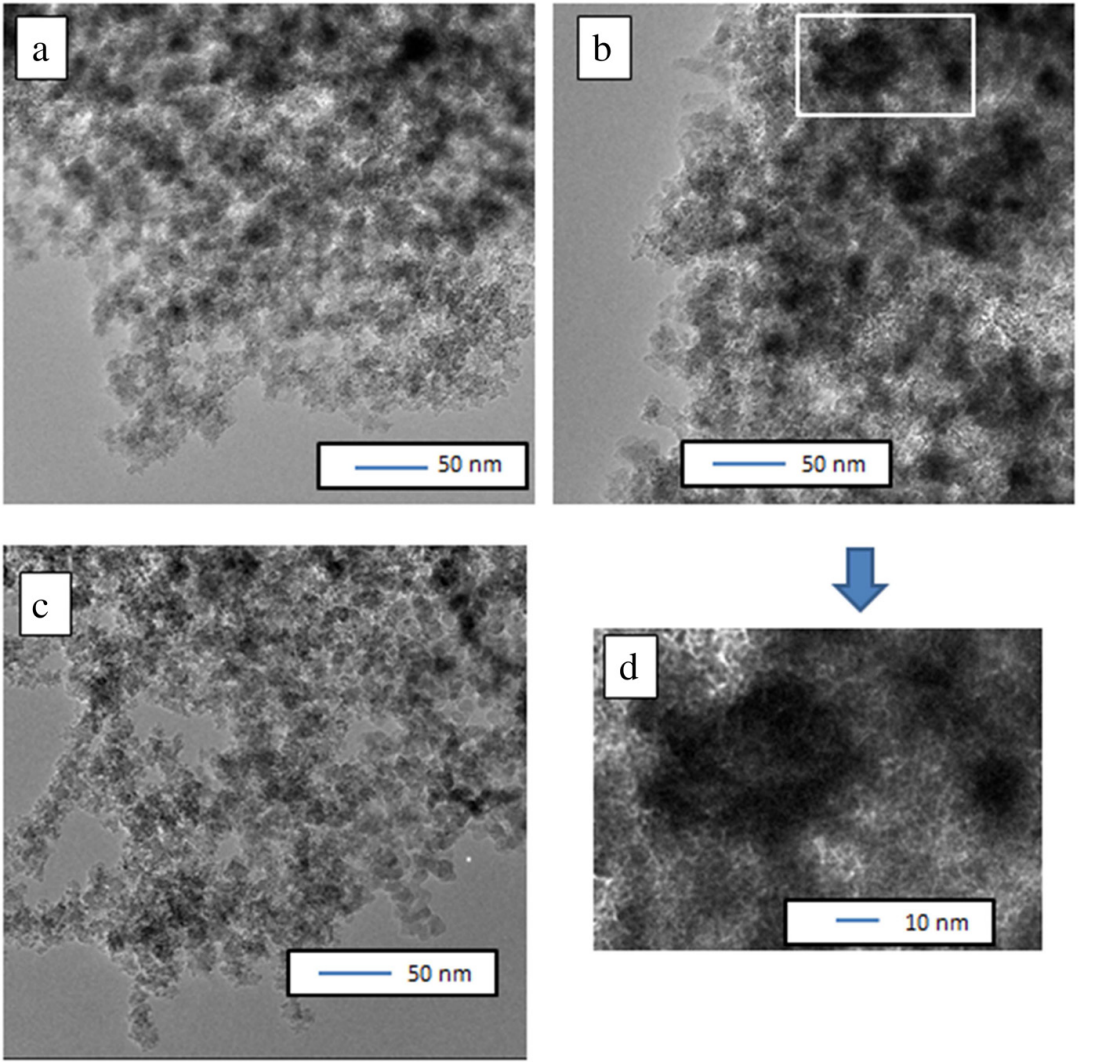
TEM micrographs of TiZrSi1 (**a**) and TiZrSi2 (**b**, **d**) samples calcined at 550 °C and initial SiO_2_ (**c**)

HRTEM images of TiO_2_–ZrO_2_/SiO_2_ nanocomposites (Fig. 2) show the formation of titania–zirconia particles (dark structures) at the silica surface (light structures). The aggregated structures of grafted oxides varying between 15 and 50 nm in size are well observed for TiZrSi1–2. Composites look like more compacted than initial silica. Therefore, contribution of macropores increases (Fig. 1b), as well as the total pore volume *V*_p_ and *V*_macro_ (Table 1) as an increased part of the empty volume (*V*_em_ = 1/*ρ*_b_−1/*ρ*_0_, where *ρ*_0_ and *ρ*_b_ are the true density of oxide nanoparticles and bulk density of the powder, respectively), in the powders. Note that any treatment or modification of fumed silica results in a decrease in the value of *V*_em_, i.e., the value of *ρ*_b_ increases, and sometimes the value of *V*_p_ increases, despite a decrease in *V*_em_, because nitrogen can fill only a portion of macropores even at *p*/*p*_0_ → 1 [28].

### X-Ray Powder Diffraction Analysis

**Fig. 3:**
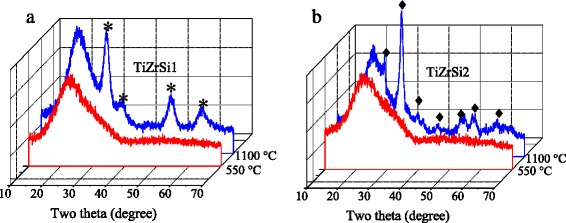
XRD patterns of TiZrSi1 (**a**) and TiZrSi2 (**b**) samples calcined at 550 and 1100 °C. *Asterisks* correspond to *t*-ZrO_2_ and *black diamonds* correspond to ZrTiO_4_

XRD analysis of TiO_2_–ZrO_2_/SiO_2_ containing different amounts of TiO_2_ (Fig. 3) shows that the samples TiZrSi1 and TiZrSi2 calcined at 550 °С are amorphous. A broad peak in the range of 20–23° is due to amorphous silica [29, 30]. Calcination at 1100 °C resulted in the appearance of crystalline phases: *t*-ZrO_2_ (PDF-ICDD 80-0965) for TiZrSi1 and ZrTiO_4_ (PDF-ICDD 74-1504) for TiZrSi2 (Table 2). For TiZrSi1, there are four sharp peaks at 30.5°, 35.3°, 50.4°, and 60.2°, which can be attributed to diffraction planes (111), (200), (220), and (311) of tetragonal zirconia (No. 79–1771). TiZrSi2 is characterized by peaks at 25.3°, 30.5°, 35.3°, 42.1°, 50.4°, 53.8°, and 61.4°, which can be assigned to the planes (101), (111), (200), (211), (202), (204), and (311) of crystalline ZrTiO_4_. The broad diffraction peaks indicated a small size of crystallites that signifies the influence of the silica substrate preventing consolidation of nuclei of grafted oxides. The average size of crystallites (*D*_cr_) revealed a nominal increase with increasing titania content (Table 2). Thus, the use of fumed silica as the inert substrate results in the formation of small nanocrystallites of grafted oxides, only.

**Table 2:** Characteristics of TiO_2_–ZrO_2_/SiO_2_ composites calcined at different temperatures

Sample ID	Composition	С_ZrO2_ (wt.%)	С_TiO2_ (wt.%)	С_SiO2_ (wt.%)	D_cr_ (nm)	
					550 °С	1100 °С
SiO_2_	SiO_2_	–	–	100		a
TiZrSi1	TiO_2_–ZrO_2_/SiO_2_	10	3	87	a	4 (b)
TiZrS2	TiO_2_–ZrO_2_/SiO_2_	10	10	80	a	7 (c)

*a* amorphous, *b t*-ZrO_2_, *c* ZrTiO_4_

### Raman Spectroscopy

**Fig. 4:**
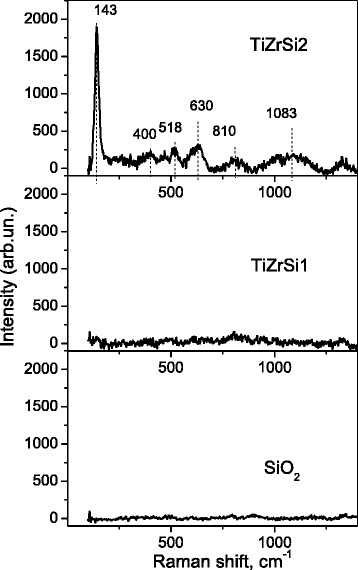
Raman spectra for initial silica and composite TiZrSi1 and TiZrSi2

Raman spectroscopy (Fig. 4) allows to get more information on the sample structure, composition effects, features of phase transition, and the quantum size effect. Fumed silica does not show any Raman features, as reported in the literature [31, 32]. It is known that zirconia exists as three polymorphs: monoclinic (*m*-ZrO_2_), tetragonal (*t*-ZrO_2_), and cubic (*c*-ZrO_2_). However, no Raman bands at 280, 316, 462, and 644 cm^−1^ due to tetragonal ZrO_2_ [33] or at 615 and 638 cm^−1^ due to monoclinic ZrO_2_ [34] are observed.

Additionally, no Raman bands at 148, 263, 476, and 550 cm^−1^ due to three-dimensional amorphous zirconia [35] are detected. For each sample, the spectra were recorded at several points, and no shift in the band position or differences of width were observed. This observation clearly reveals that all of the samples are mostly in a homogeneous state. For sample TiZrSi1, characteristic Raman bands are not observed. However, for sample TiZrSi2, the well-resolved Raman peaks at 143, 400, 500, 518, 630, 810, and 1083 cm^−1^ are observed. Some of these bands are specific to anatase [36] at 143 cm^−1^ (E_g_, very strong), 197 cm^−1^ (E_g_), 396 cm^−1^ (B_1g_), 514 cm^−1^ (A_1g_, B_1g_), and 637 cm^−1^ (E_g_).

The obtained Raman spectrum is well correlated with the data for ZrTiO_4_ [37, 38]. It was noted [33] that the variations in broad bands at 148 (E_g_), 401 (B_1g_), 522 (A_1g_ or B_1g_), and 648 (E_g_) cm^–1^ are characteristic for anatase depending on the ratio TiO_2_:ZrO_2_ in films, but no other bands characteristic for other polymorphs were found. According to [37], the degree of line broadening in a peak at 815 cm^−1^ as probing local microstructure was chosen because this peak did not overlap with other peaks and exhibited a pronounced change in the degree of line broadening.

Thus, it can be seen that anatase is formed only at relatively high concentration of TiO_2_ in the composite, whereas at a low concentration of TiO_2_, the amorphous titania is observed. Based on the presence of the background at the location of line E_g_(1) for TiZrSi2, an amorphous phase is also present. The intensity of the Raman bands depends on several factors including grain size and morphology [38]. A strong increase in line E_g_(1) background at the presence of small (2–3 nm) crystallites was also noted previously [39]. Peak E_g_(2) near 197 cm^−1^ has a very low intensity and in our composites is not observed.

The absence of any other Raman features providing inference that silica does not form any compound with titania and zirconia is in line with XRD observations.

### Surface Characterization by XPS

**Fig. 5:**
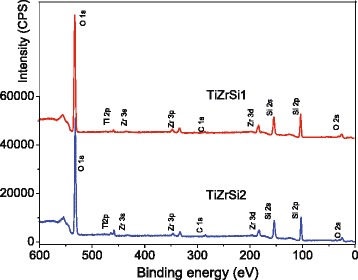
Wide XPS spectra: TiZrSi1 and TiZrSi2

Formation of chemical bonds between components in ternary oxides was investigated using the XPS method (Fig. 5). Two main peaks for silicon (Si 2s and Si 2p), two peaks for zirconium (Zr 3p and Zr 3d), and only one main peak for titanium (Ti 2p doublet) were detected in the spectra (Fig. 5). For all the samples, analysis of the 1s line of the carbon showed that the states with a binding energy within 284.7–290.8 eV are formed by a variety of carbon bonds of surface hydrocarbon contamination of samples [40].

**Fig. 6:**
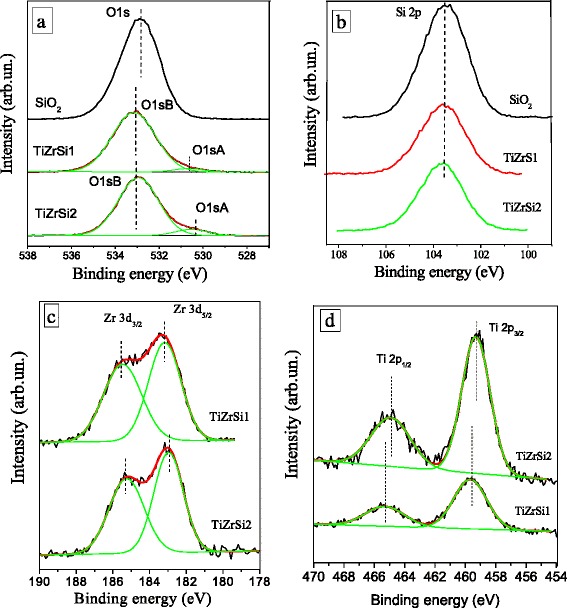
Detailed XPS of O 1s (**a**), Si 2p (**b**), Zr 3d (**c**), and Ti 2p (**d**) initial silica (Si 2p, O 1s) and TiO_2_–ZrO_2_/SiO_2_ at different contents of TiO_2_ (TiZrSi1 and TiZrSi2) calcined at 550 °C

For the analysis of the chemical state of elements forming nanolayers TiO_2_–ZrO_2_/SiO_2_, the following line core levels Si 2p, O 1s, Zr 3d, and Ti 2p were selected. The detailed XPS spectra of oxygen for silica and ternary oxide samples are compared (Fig. 6a). In oxygen O 1s region, one can see that the positions of O 1s are slightly shifted in samples TiZrSi1 and TiZrSi1 compared to the initial silica. For TiO_2_–ZrO_2_/SiO_2_, the O 1s peak can be divided into two bands O 1s A and O 1s B, and the ratio of these components depends on the content of titania (Fig. 6 and Table 3). The appearance of the O 1s peak at lower energy is due to the effects of TiO_2_ and ZrO_2_ with a large displacement of the electron density to the O atoms than that in silica.

**Table 3:** XPS core-level binding energy values (eV) for samples studied

Sample ID	O 1s	Si 2p	Zr 3d_5/2_	Zr 3d_3/2_	Ti 2p_3/2_	Ti 2p_1/2_
SiO_2_	532.90	103.52	–	–	–	–
TiZrSi1	530.5	103.6	183.3	185.6	459.6	465.4
	533.2					
TiZrSi2	530.5	103.7	183.1	185.4	459.3	465.0
	533.0					

The binding energy of the Si 2p peak ranged between 103.5 and 103.7 eV (Fig. 6b) that are consistent with the values reported in the literature [40]. The weak intensity of the spectra with large peak widths in case of TiZrSi1 and TiZrSi1 samples indicates that silica is not easily accessible at the surface due to the presence of titania–zirconia layers.

The Zr 3d_5/2_ and Ti 2p_3/2_ peaks (Fig. 6c, d) correspond to the binding energy of 183.1–183.3 and 459.3–459.6 eV, respectively, which represent the fully oxidized zirconium ion Zr^4+^ and titanium ion Ti^4+^ [39]. Such binding energies can be attributed both to the individual metal oxides [39] and to ZrTiO_4_ [41, 42]. The observed positive shifts of the peaks Ti 2p_3/2_ and Ti 2p_1/2_ (Fig. 6d) relatively to the peaks in individual titania (458.7 and 464.7 eV) [40] may testify the formation of the Ti–O–Zr bonds. The displacement was observed [43] for the mixed triple films TiO_2_/ZrO_2_/SiO_2_. Note that during mixed oxide formation, the inhibitive influence on the growth and agglomeration of the individual phases of the components occurs due to the formation of the Ti–O–Zr bonds. In the investigated samples, the shift of the Ti 2p_3/2_ peak relatively to pure TiO_2_ is larger for TiZrSi1 at smaller content of TiO_2_ than for TiZrSi2 with a high content of TiO_2_. This fact shows that at increasing TiO_2_ content in the ternary oxide, the number of the Ti–O–Zr bonds decreases, i.e., at higher content, TiO_2_ forms a separate phase, while at lower content it forms TiO_2_–ZrO_2_ mixed oxide.

### Particle Size Distribution

**Fig. 7:**
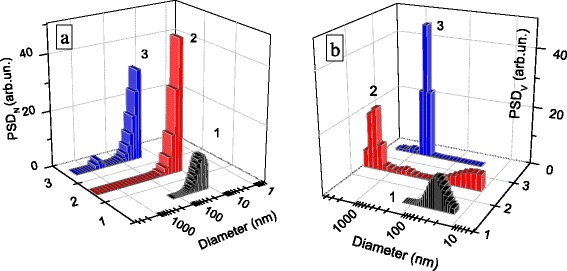
PSD related to **a** particle number and **b** volume for silica and composites after sonication (3 min) of the aqueous suspensions (*C* = 0.1 wt.%) of initial SiO_2_ (*1*), TiZrSi1 (*2*), and TiZrSi2 (*3*)

The degree of aggregation/agglomeration of nanoparticles depends on their characteristics and interactions with the dispersion medium. The initial silica is characterized by nearly monomodal particle size distribution (PSD) with a maximum at 21 nm (Fig. 7a, curve 1).

However, the PSD for composites is bimodal with two peaks with respect to the particle number (Fig. 7a) and particle volume (Fig. 7b). The PSDs for TiZrSi1 and initial silica are similar, while for TiZrSi2, the aggregates are characterized by larger sizes ~500 nm. Note that there is a tendency of increasing particle size with increasing TiO_2_ content in the composites (Fig. 7, curves 2–3). The increase of the average particle size in aqueous suspensions can be associated as with a change in particle size during the formation of a new phase of ZrO_2_/TiO_2_ during the synthesis and also with influence of changes in surface structure and related electrokinetic properties of the oxide composites on the aggregation processes in an aqueous medium.

## Conclusion

In the present study, highly disperse silica-supported titania–zirconia nanocomposites were synthesized by a liquid-phase method. The samples were examined using a set of techniques after their calcination at 550 and 1100 °C. The structural characteristics (phase composition, average size of crystallites) of the materials affected by pre-heating were determined from the XRD data. The XRD measurements indicated the presence of ZrTiO_4_ and anatase in TiZrSi2 and tetragonal zirconia in TiZrSi1 calcined at 1100 °C. The TiZrSi1 and TiZrSi2 samples calcined at 550 °С were XRD amorphous. The crystallinity slightly increased with increasing titania content in nanocomposites. There is no indication of compound formed with silica and titania or zirconia. The analysis of the nitrogen adsorption–desorption data and HRTEM indicates that the grafting new oxide phases changes the textural characteristics of the powders. The incremental pore size distribution functions revealed the existence of mainly meso/macroporosity of aggregates of initial silica and mainly macroporosity of TiO_2_–ZrO_2_/SiO_2_ nanocomposites. The HRTEM images show the presence of well-dispersed Zr–Ti–oxide nanocrystallites ~15–50 nm in size on the amorphous silica matrix. In line with XRD results, Raman spectra show that silica did not form any compound with titania or zirconia. The XPS results reveal that O 1s, Si 2p, Zr 3d, and Ti 2p core-level photoelectron peaks are sensitive to the phase composition of TiO_2_–ZrO_2_/SiO_2_ nanocomposites. Moreover, XPS measurements show that Zr and Ti ions are present in their highest oxidation states (+4). The shift of the peaks indicates the possible formation of titanium–zirconium mixed oxide. A tendency of increasing particle size with increasing TiO_2_ content in the composites was detected accordingly to the PSD characterization in the aqueous media.
